# The radiological and histological investigation of the dental follicle of asymptomatic impacted mandibular third molars

**DOI:** 10.1186/s12903-022-02681-6

**Published:** 2022-12-26

**Authors:** Kuncai Li, Wei Xu, Tiejun Zhou, Junliang Chen, Yun He

**Affiliations:** 1 Department of Oral and Maxillofacial Surgery, The Affiliated Stomatological Hospital of Southwest Medical University, Luzhou, China; 2 Oral and Maxillofacial Reconstruction and Regeneration of Luzhou Key Laboratory, Luzhou, China; 3 Department of Pathology, Affiliated Hospital of Southwest Medical University, Luzhou, China

**Keywords:** Third molars, Tooth extraction, Cone beam computed tomography, Dental follicle, Odontogenic cyst

## Abstract

**Objectives:**

The indication for removal of asymptomatic fully impacted third molars is still controversial. In this study, radiological and histological investigation of the dental follicle of asymptomatic impacted mandibular third molars was performed, aiming to provide a reference for clinical prophylactic extraction of these teeth.

**Methods:**

Patients with impacted mandibular third molars were included and the maximum width of the dental follicle around the crown was measured in horizontal, sagittal and coronal sections by cone beam computed tomography. The dental follicles were stained with haematoxylin-eosin, analysed by a pathologist and classified as normal, inflammatory or cystic. A Chi-squared test was used to analyse the association of the incidence of inflammation and cysts with the clinical variables of the impacted mandibular third molars.

**Results:**

Thirty-seven samples were normal dental follicles; 52 samples showed inflammatory infiltration with an incidence of 57.14%; 2 samples with a maximum dental follicle width of 2–3 mm were diagnosed as odontogenic cysts, and the incidence was 2.20%. There was no significant difference in the incidence of inflammatory and cystic dental follicles between males and females, or between different age groups (*P* > 0.05). With an increase of the maximum width of the dental follicle, there was a rise in the incidence and degree of infiltration of chronic nonspecific inflammation.

**Conclusion:**

Asymptomatic impacted mandibular third molars tend to be extracted, especially for teeth with a 2–3 mm maximum width of the dental follicle on radiological examination.

## Background

Third molars are normally the last teeth to erupt [1, 2]. They are prone to impaction and lead to several problems, such as pericoronitis, caries and temporomandibular disorders (TMD), due to space limitations, physical barriers or other anatomical factors [3–5]. Therefore, the extraction of impacted third molars is the most common surgery in dentoalveolar surgery. However, the indication for removal of asymptomatic fully impacted third molars is still controversial [4–7]. On one hand, the extraction of fully impacted third molars might be associated with multiple complications such as bleeding, pain, swelling and inferior alveolar nerve (IAN) injury [8–11]. Therefore, some patients might be reluctant to deal with asymptomatic fully impacted third molars. On the other hand, the absence of clinical symptoms does not imply the absence of illnesses. For example, inflammatory, cystic or neoplastic tumours might be found in the dental follicle tissue surrounding these third molars even with no clinical evidence of disease [4, 7, 12–14].

The term dental follicle has been traditionally used to refer to the residual connective and epithelial tissue attached to the crown of unerupted teeth. It plays a critical role in tooth and periodontal tissue development, as well as in tooth eruption [15] It denotes a tissue remnant after the tooth, cementum, alveolar bone and periodontal ligament have formed [15, 16]. Under normal circumstances, the dental follicle disappears after tooth eruption. However, in cases of unerupted teeth, the dental follicles remain in the jawbones, even if the teeth are fully developed [15]. Radiologically, a dental follicle is characterized by thin semicircular radial light transmission around unerupted or impacted teeth, and an enlarged or asymmetric dental follicle can be occasionally observed [17].

Radiological examination should be carried out before extraction of impacted teeth, to observe the tooth morphology and adjacent tissues such as the pericoronal dental follicle, adjacent tooth, inferior alveolar nerve and so on. The most commonly used methods are panoramic radiograph and cone beam computed tomography (CBCT). A panoramic radiograph presents a two-dimensional image. In addition, CBCT offers a high number of pixels, high definition, high resolution, quick imaging speed and low radiation, as well as the ability to display tomographic and three-dimensional pictures from any angle [18, 19].

In this study, the width of the dental follicle surrounding the crown of asymptomatic impacted mandibular third molars was measured using CBCT, and histopathological analysis of the dental follicle was performed by haematoxylin-eosin (HE) staining. The study aims to reveal the radiological and histological characteristics of the dental follicle surrounding asymptomatic impacted mandibular third molars and to offer a reference for clinical prophylactic extraction of asymptomatic impacted mandibular third molars.

## Materials and methods

This study was approved by the Institutional Ethics Committee of the Affiliated Stomatological Hospital of Southwest Medical University (certificate number 20210525001) and conducted in accordance with the Declaration of Helsinki. All procedures in this study were carried out with the full understanding and written consent of the subjects. Informed consent was obtained from all included patients in the ethics subsection of declaration section too.

### Patient selection

From July 2021 to February 2022, patients with impacted mandibular third molars and who needed surgery to remove the teeth were recruited from the Department of Oral and Maxillofacial Surgery at the Affiliated Stomatological Hospital of Southwest Medical University (Luzhou, China). Informed consent was obtained from all included patients. The inclusion criteria were on the basis of clinical and radiological examination: (1) The mandibular third molars were intraorally invisible and completely covered with mucosa and bone. (2) There were no clinical symptoms associated with the impacted mandibular third molars. (3) The roots of the teeth were completely formed. This criterion was used because the dental follicles of teeth with undeveloped roots are still forming periodontal tissue and might manifest larger areas of radiolucency in images [15, 20]. (4) Patients had good general health and did not take any type of medication. The exclusion criteria were: (1) The roots of the teeth were not completely formed. (2) The maximum width of the radiolucent area of the dental follicle around the crown was greater than or equal to 3 mm. This was included because an area larger than that is regarded as a radiographically pathologic state [12, 21]. (3) Patients with uncontrolled systematic disease. Basic information of the patients (gender, age, etc.) was recorded.

### Radiographic analysis

All patients were scanned with a KODAK 9500 CBCT (Carestream Health, Rochester, NY, USA) at 86 kVp, 10 mAs and 10.8 s exposure, with a resolution of 0.2 mm per slice. The three-dimensional reconstruction images were obtained by multiplanar reconstruction (MPR). Image data were evaluated using the built-in CS 3D Imaging Software 3.5 (Carestream Health, Rochester, NY, USA).

**Fig. 1:**
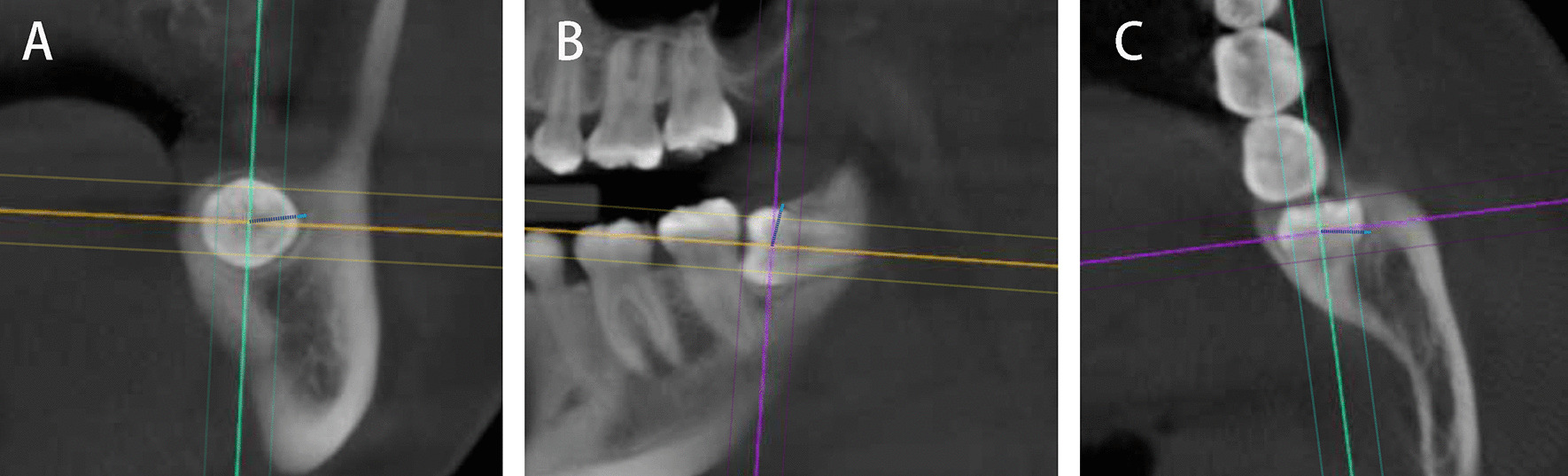
Measurement of the maximum width of the dental follicle around the crown by CBCT. **A** The measurement in coronal section. **B** The measurement in Sagittal section. **C** The measurement in horizontal section. The blue line indicates the width of the dental follicle

Measurement was performed on three sections: a horizontal section and longitudinal sagittal section through the long axis of the tooth, and a coronal section through the middle of the crown. The three sections were reconstructed at intervals of approximately 10 mm to ensure that the sections contained the whole crown. The maximum width of the radiolucent area of the dental follicle around the crown was measured in the three sections, respectively, with the intersection of vertical lines as the starting point (Fig. 1). These steps were performed in triplicate by the same investigator. The mean values on the three planes were calculated and grouped into 0–1 mm, 1–2 mm and 2–3 mm. The reason for choosing this measurement method is that it can be repeated and stably applied to every tooth in the CBCT image, ensuring the repeatability, accuracy and stability of the measurement.

### Surgical procedure

**Fig. 2:**
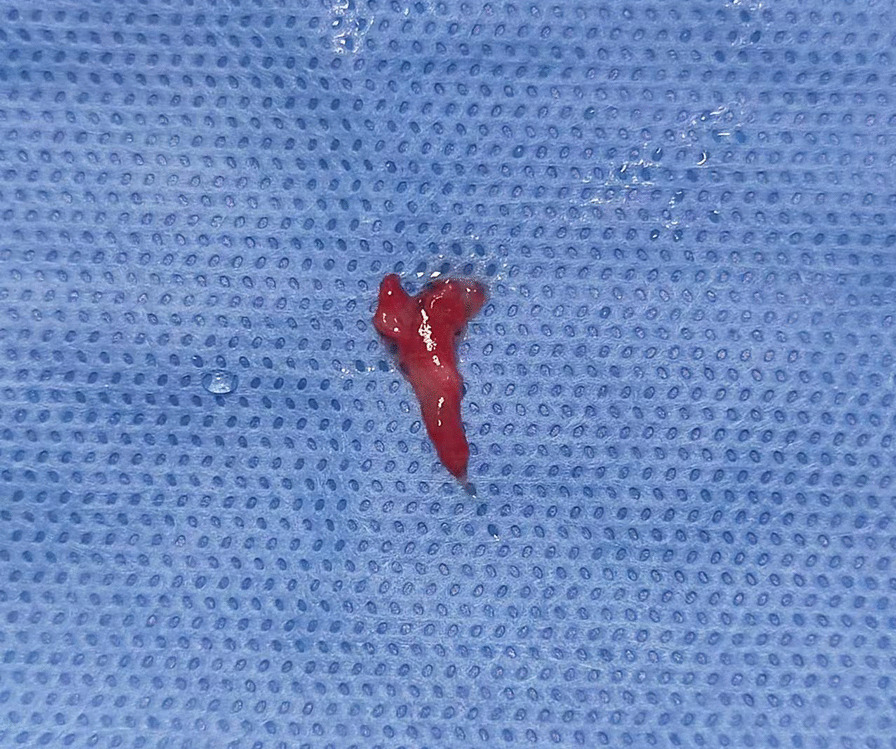
Dental follicle of the asymptomatic impacted mandibular third molars

According to the inclusion criteria, the patients underwent surgical removal of the impacted mandibular third molars. Local anaesthesia was induced with 2% lidocaine and 1% articaine. Minimally invasive extraction technology was used to extract the teeth. A contra-angle high-speed hand-piece with a tungsten carbide bur was used for boning and tooth sectioning under irrigation. After tooth extraction, a curette and haemostat were used to obtain the dental follicle tissue for histopathological analysis (Fig. 2).

### Histopathological analysis

The specimens were immediately fixed in a 10% formalin solution for 48 h, dehydrated with an increasing concentration of alcohol (70%, 80%, 90%, 95% and 100%) and embedded in paraffin. The paraffin blocks were sectioned with the aid of a microtome to obtain slices with a thickness of 5 μm. The slices were then stained by HE and visualized by an orthographic optical microscope (BX53, Olympus, Japan).

The slices were submitted for histological examination and diagnosed by a pathologist who had undergone a training and calibration exercise. The dental follicles were classified as normal or pathological according to the histological findings.

1. Normal dental follicle. The presence of fibrous connective tissue with remnants of reduced enamel epithelium in the fibrous connective tissue layer and the absence of an epithelial lining, with or without islets or strings of odontogenic epithelium, amorphous basophilic mineralized material and/or areas of extravasation of red cells [7, 12].
2. Pathological dental follicle. There were two categories. One was chronic nonspecific inflammatory tissue, which showed inflammatory infiltration of the dental follicle with or without pseudoepitheliomatous hyperplasia (PEH), papillomatous hyperplasia of the epithelium, mucoid degeneration, fibroplasia, tissue calcification and epithelial hyperplasia. The other was odontogenic cysts, in which Rushton bodies among the epithelium, and a dense, fibrous connective tissue wall lined by a few layers of stratified squamous epithelium could be observed [7, 22].

### Statistical analysis

Data were statistically analysed using the IBM statistical package SPSS 22.0 (IBM Co., Chicago, IL, USA). The incidence of normal follicles and the two kinds of pathological dental follicles was calculated and recorded as a percentage. A Chi-squared test was used to analyse the association of the incidence of inflammation and odontogenic cysts with the clinical variables (gender, age and the maximum width of the dental follicle) of the impacted mandibular third molars. A *P* value < 0.05 was considered indicative of statistical significance.

## Results

### Characteristics of patients

Thirty-nine of the samples were from male patients and 52 were from female patients (Table 1). Patient age ranged from 18 to 42 years, with a mean value of 23.95 ± 4.20 years.

**Table 1:** Characteristics of the included patients

Variables	N (%)	Normal dental follicle	Pathological dental follicle	
			Chronic nonspecific inflammatory	Odontogenic cyst
Sex				
Male	39 (42.86%)	15	24	0
Female	52 (57.14%)	22	28	2
Age (years)				
≦25	59 (64.84%)	24	34	1
>25	32 (35.16%)	13	18	1
Maximum width of the dental follicle (mm)				
0–1	37 (40.66%)	18	19	0
1–2	45 (49.45%)	19	26	0
2–3	9 (9.89%)	0	7	2
Total	91	37	52	2

### Results of radiographical analysis

As for the maximum width of the dental follicle, 37 samples were less than 1 mm, 45 samples were within 1–2 mm and 9 samples were within 2–3 mm (Table 1).

### Results of histopathological analysis

**Fig. 3:**
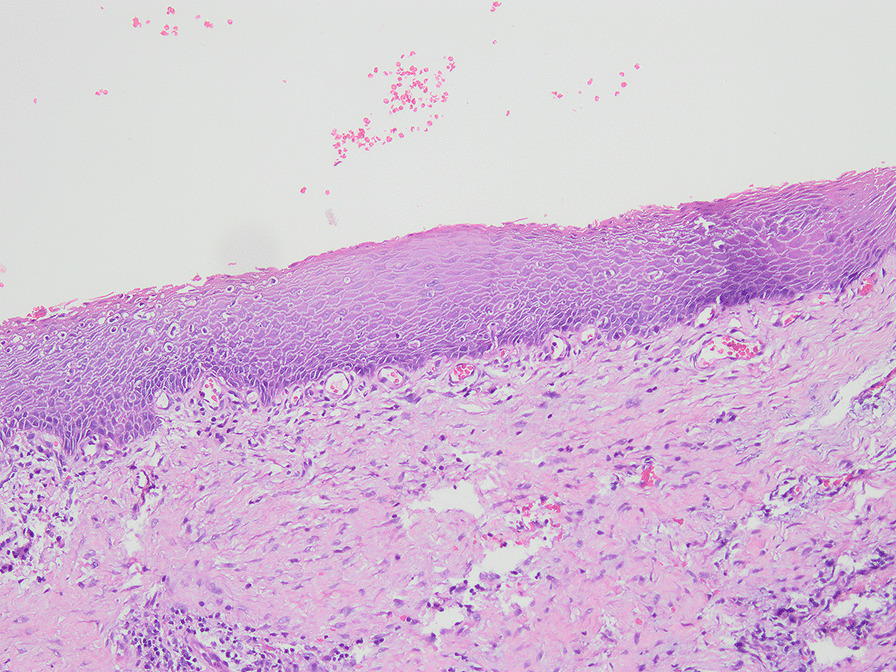
Normal dental follicle without epithelial exfoliation (×100)

**Fig. 4:**
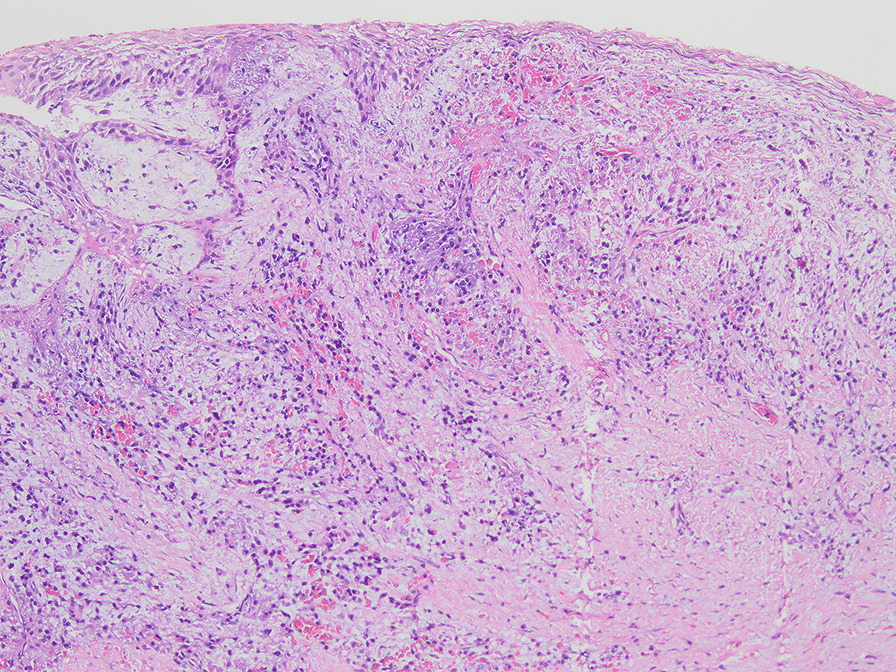
Inflammatory infiltration of the dental follicle (×100)

**Fig. 5:**
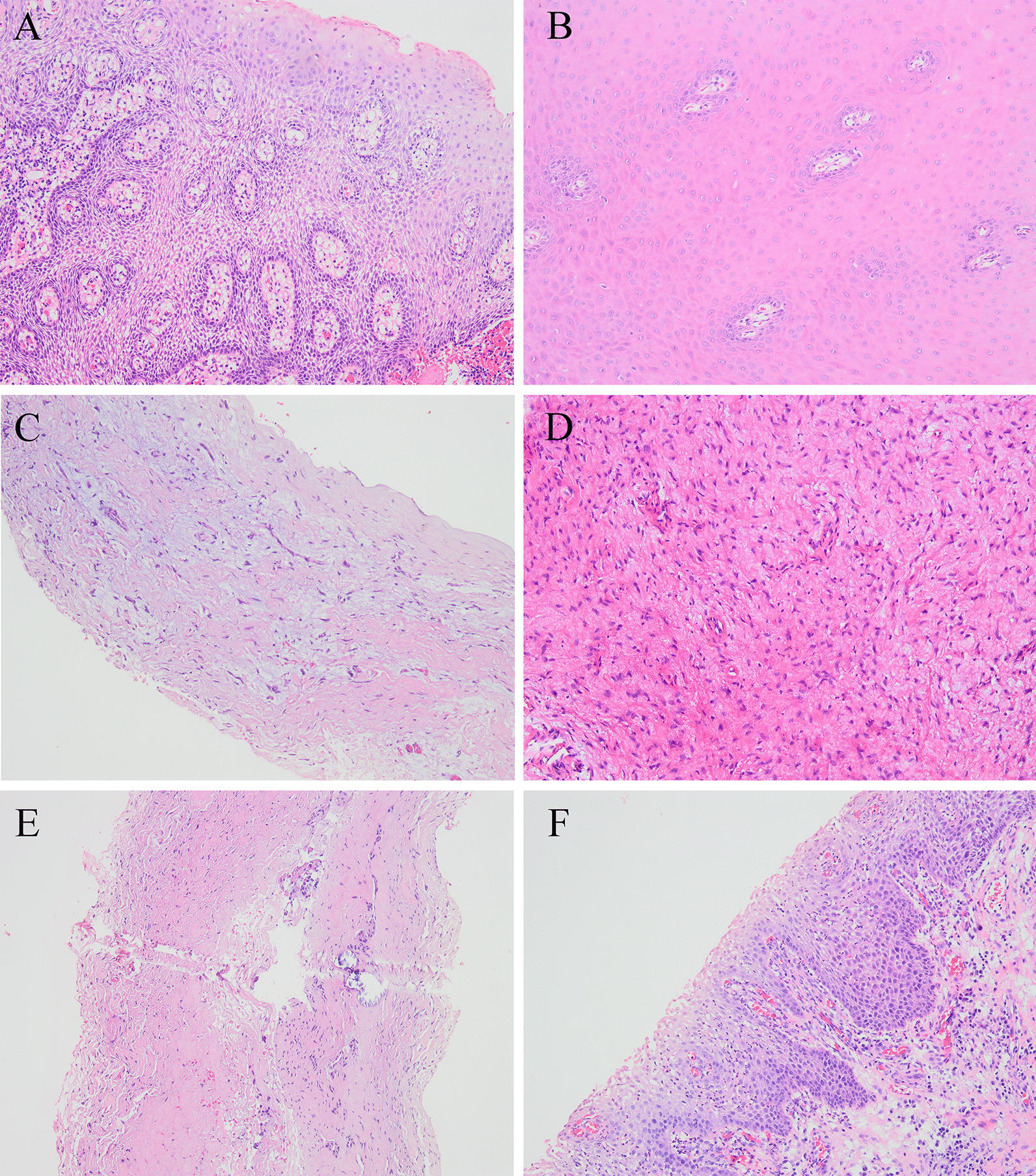
Pathological features caused by inflammatory stimulation (×100). **A** PEH, **B** Papillomatous hyperplasia of epithelium, **C** Mucoid degeneration, **D** Fibroplasia, **E** Tissue calcification, **F** Epithelial hyperplasia

**Fig. 6:**
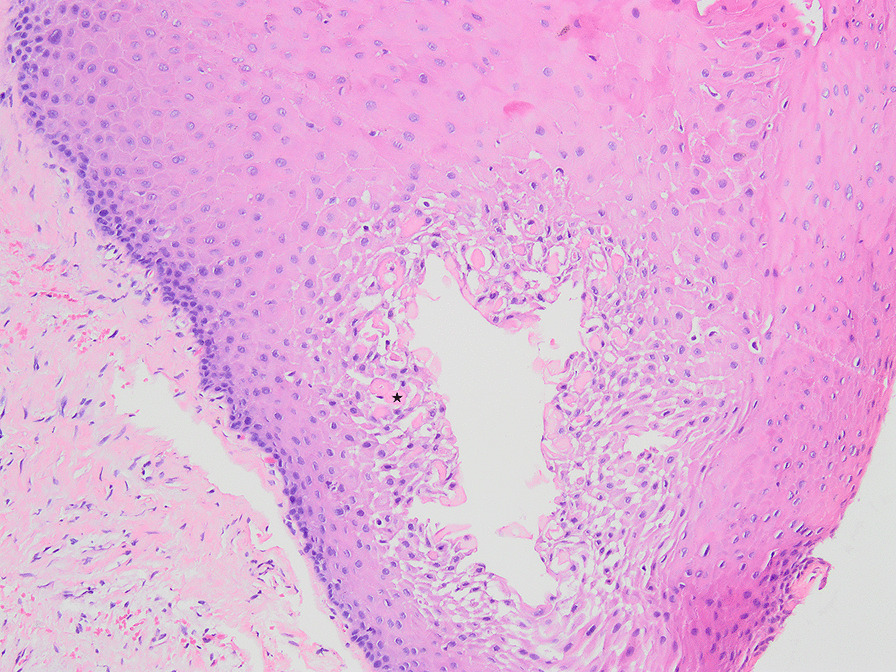
Odontogenic cyst of the dental follicle (×100). Rushton body indicated by ★

Histologically, 37 samples were normal (Fig. 3), and 54 samples showed pathological dental follicles, with an incidence of 59.34%. Fifty-two samples showed inflammatory infiltration in connective tissue or epithelial tissue (Fig. 4), with an incidence of 57.14%. Moreover, some of them showed PEH, papillomatous hyperplasia of the epithelium, mucoid degeneration, fibroplasia, tissue calcification and epithelial hyperplasia caused by inflammatory stimulation (Fig. 5). In 2 samples, with an incidence of 2.20%, Rushton bodies in epithelial tissues surrounded by multiple layers of squamous epithelium were observed, and they were diagnosed as odontogenic cysts (Fig. 6).

### Gender and histopathology

Fifty-two samples were chronic nonspecific inflammatory tissue, 24 from males (61.54%) and 28 from females (53.85%). There was no significant difference in the incidence of inflammation between males and females (*P* > 0.05) (Table 2). Odontogenic cysts were observed in 2 impacted teeth of females. There was no significant difference in the incidence of cysts between males and females (*P* > 0.05).

**Table 2:** Incidence of inflammation and odontogenic cysts in the clinical variables (gender, age and the maximum width of the dental follicle) of the impacted mandibular third molars

Variables	Total	Normal dental follicle		Pathological dental follicle					
		n	%	Chronic nonspecific inflammatory			Odontogenic cyst		
				n	%	*p*	n	%	*p*
Sex									
Male	39	15	38.46	24	61.54	0.668	0	0.00	0.514
Female	52	22	42.31	28	53.85		2	3.85	
Age (years)									
≦25	59	24	40.68	34	57.63	1.000	1	1.69	1.000
>25	32	13	40.63	18	56.25		1	3.12	
Maximum width of the dental follicle (mm)									
0–1	37	18	48.64	19	51.35	0.052	0	0.00	0.001
1–2	45	19	42.22	26	57.78		0	0.00	
2–3	9	0	0.00	7	77.78		2	22.22	

### Age and histopathology

Among the 52 inflammatory samples, 34 samples were from patients less than or equal to 25 years old and 18 samples were from patients more than 25 years old, with an incidence of 57.63% and 56.25%, respectively. There was no significant difference in the incidence of inflammation between the two age groups (*P* > 0.05) (Table 2). There was one case of odontogenic cysts in each of the two age groups. There was no significant difference in the incidence of odontogenic cysts between the two age groups (*P* > 0.05).

### Maximum width of the dental follicle and histopathology

With an increase of the maximum width of the dental follicle, there was a rise in the incidence and degree of infiltration of chronic nonspecific inflammation, and the incidence of chronic nonspecific inflammation in the 2–3 mm group was significantly higher than that in the 0–1 mm and 1–2 mm groups (*P* < 0.05). However, there was no significant difference between the 0–1 mm and 1–2 mm groups. Odontogenic cysts were only found in the 2–3 mm group (Table 2). In the 0–1 mm maximum dental follicle width group, 19 samples showed chronic nonspecific inflammation and the incidence was 51.35%. In the 1–2 mm maximum dental follicle width group, 26 of the samples showed chronic nonspecific inflammation and the incidence was 57.78%. Pathological changes were observed in all samples in the 2–3 mm maximum dental follicle width group; 7 samples showed chronic nonspecific inflammation and the incidence was 77.78%; 2 samples were diagnosed as odontogenic cysts (Table 2), an incidence of 22.22% which was significantly higher than in the other groups (*P* < 0.05).

## Discussion

Extraction of impacted third molars has become one of the most common and delicate procedures performed in dental clinics. It is reported that with an increase of age, the difficulty of extracting impacted teeth will increase, as well as the risk of postoperative complications, so it is necessary to extract impacted teeth in time [23, 24]. However, the indication of prophylactic extraction of asymptomatic impacted third molars has still been controversial for many years due to the imbalance between the advantages and disadvantages associated with the surgical procedure [25]. On the one hand, the extraction of impacted third molars might be relatively difficulty, and associated with multiple complications. The coronectomy and orthodontic-assisted extraction are also proposed by some doctors in order to reduce the trauma and the incidence of IAN injure [26, 27]. However, the former has the risk of infection and second operation, while the latter has a long treatment cycle. On the other hand, Shin et al. [13] evaluated the prevalence of cysts or tumours associated with impacted mandibular third molars and found that the incidence of cysts or tumours increased with age, and supposed that preventive extraction needs more investigation and research. Palma et al. [12] reported that the pathological changes of dental follicles around mandibular impacted third molars may appear as inflammation, dental cysts and so on, and advocated the removal of asymptomatic impacted teeth. Yildirim et al. [28] found that asymptomatic impacted third molars would manifest cystic changes and considered that they should be extracted before pathological changes occur. However, the study of Steed et al. [3] showed that the clinical and pathological data do not justify the extraction of asymptomatic third molars.

Besides the histopathological analysis of asymptomatic third molars, some scholars also perform radiological analysis. The dental follicle surrounding the tooth is interpreted in the radiograph as pericoronal radiolucency. The width of this radiolucent area is one of the importance approaches to differentiate between a normal dental follicle and an abnormal one. Tambuwala et al. [17] and Baykul et al. [29] analysed the pericoronal radiolucent areas (dental follicles) of impacted mandibular molars using panoramic radiographs. However, panoramic radiographs are two-dimensional overlapping images, and interpretation of the width of the pericoronal radiolucent area might be not accurate [30, 31]. Therefore, CBCT was used in this study, as a three-dimensional imaging detection method to accurately measure the maximum width of the dental follicle. The study aims to combine radiological and histological methods to investigate the dental follicles of asymptomatic impacted mandibular third molars.

There were three histopathologic situations of the dental follicle in this study, normal, chronic nonspecific inflammation and odontogenic cyst. The results indicate that 59.34% of samples were pathological dental follicles. This percentage is similar to those reported in studies by other authors [7, 29]. There was no significant difference in the incidence of inflammation and odontogenic cysts of the dental follicle around impacted mandibular third molars between males and females, or between patients less than 25 years old and those older than 25 years old.

Inflammation was the most frequent pathological alteration in the dental follicles analysed. A total of 52 samples with an incidence of 57.14% were diagnosed as chronic nonspecific inflammatory tissue in our study. Inflammation of the dental follicles around asymptomatic teeth might be due to two reasons [21]. Firstly, many of these teeth may be in a state of eruption, although the process will not be completed. The process of tooth eruption is usually accompanied by inflammation, which results from the infiltration of oral antigens into the wider intercellular space between the reduced enamel organs and the epithelial cells of the oral epithelium [32]. Secondly, asymptomatic impacted teeth also might communicate with the oral environment through the periodontal tissue of the adjacent teeth. Inflammation factors could infiltrate into the dental follicle through the periodontal tissue of the adjacent teeth. At the same time, inflammation in the dental follicle could influence the health of soft and hard tissue surrounding the tooth, such as periodontal tissue, gingiva, bone and so on. The results of this study indicate that with an increase of the maximum width of dental follicles, the incidence of inflammation is increased, and the degree of inflammatory infiltration is also increased. This illustrates that the maximum width of the dental follicle is positively correlated with the incidence of inflammation in the dental follicle.

Odontogenic cysts are epithelial-lined pathologic cavities surrounded by fibrous connective tissue that originates from odontogenic tissues [33]. Odontogenic cyst enlargement may cause bone destruction and resorption or displacement of adjacent teeth, and results in irregular dentition and facial deformity and other serious consequences [34]. Periapical cysts and dentigerous cysts are frequently reported conditions in dental practice. Histopathologic examination remains the gold standard investigation. Besides, rushton bodies can only be found in odontogenic cysts [35, 36]. In this study, Rushton bodies and fibrous tissue lined by nonkeratinized stratified squamous epithelium were observed in 2 samples of the 2–3 mm dental follicle maximum width group, indicating the existence of odontogenic cysts in the dental follicle. The incidence of cysts was 2.20%.

The incidence of inflammation and odontogenic cysts in the dental follicle of impacted mandibular third molars is different in many studies. One of the reasons for this could be the difficulty in histologically differentiating a normal dental follicle from a dentigerous cyst in the initial phase [21]. Another is the different inclusion and diagnosis criteria. Tambuwala et al. [17] reported an incidence of cystic pathological changes in the dental follicle tissue of asymptomatic mandibular third molars of 11.5%. However, they did not mention the precise diagnostic criteria for cysts. The study of de Mello Palma et al. [12] indicated an incidence of inflammation and dentigerous cysts in the dental follicles of disease-free mandibular impacted third molars of 32.4% and 1.39%, respectively. The diagnostic criterion of the study was that the capsule of a dentigerous cyst is classically described in the histopathological exam as connective tissue lined with stratified squamous epithelium. Baykul et al. [29] found 50% incidence of cystic changes in radiographically normal impacted lower third molar follicles. They did not use the inclusion criterion of the roots of the teeth being completely formed. The diagnostic criteria of their study were dense fibrous connective tissue walls and several layers of stratified squamous epithelium in the specimen. However, many pathologists regard stratified squamous epithelium as an abnormal state and think that this epithelial hyperplasia is caused by inflammatory stimulation, diagnosing it as inflammation [21, 37]. Therefore, epithelial hyperplasia was diagnosed as chronic nonspecific inflammation in the present study. Moreover, the diagnostic criteria for odontogenic cysts were the observation of Rushton bodies and fibrous tissue lined by nonkeratinized stratified squamous epithelium.

Additionally, there are several limitations to the present study. The sample size was relatively limited. Further research with a larger sample size would allow verification of the results and investigation of the association between the incidence of abnormal dental follicles and other clinical variations, such as the position and classification of the impacted teeth. Secondly, regular observation and return visits could be conducted in a further study to investigate the variation of asymptomatic impacted mandibular third molars and wound healing following tooth extraction. This would allow us to evaluate the asymptomatic impacted teeth in the long run.

## Conclusion

With an increase of the maximum width of the dental follicle, the incidence of chronic nonspecific inflammation in asymptomatic impacted mandibular third molars increased. Odontogenic cysts were only found in the 2–3 mm maximum follicle width group. Therefore, asymptomatic impacted mandibular third molars tend to be extracted, especially for teeth with a maximum dental follicle width of 2–3 mm. Besides that, the status of asymptomatic impacted third molars changes dynamically and has to be inspected periodically.

## Data Availability

The datasets used and/or analyses during the current study available from the corresponding author on reasonable request.
